# Antioxidant and Anti-Inflammatory Effects of Crocin Ameliorate Doxorubicin-Induced Nephrotoxicity in Rats

**DOI:** 10.1155/2021/8841726

**Published:** 2021-02-15

**Authors:** Mona A. Hussain, Noha M. Abogresha, Ghada AbdelKader, Ranya Hassan, Eman Z. Abdelaziz, Sahar M. Greish

**Affiliations:** ^1^Department of Medical Physiology, Faculty of Medicine, Port Said University, Port Said, Egypt; ^2^Department of Medical Physiology, Faculty of Medicine, Suez Canal University, Ismailia, Egypt; ^3^Department of Human Anatomy and Embryology, Faculty of Medicine, Suez Canal University, Ismailia, Egypt; ^4^Department of Clinical Pathology, Faculty of Medicine, Suez Canal University, Ismailia, Egypt; ^5^Department of Pharmacology, Faculty of Medicine, Suez Canal University, Ismailia, Egypt; ^6^Physiology Department, School of Medicine, Badr University in Egypt (BUC), Egypt

## Abstract

Doxorubicin is a drug that belongs to the anthracycline antibiotics. Nephrotoxicity is one of the serious side effects of doxorubicin treatment. Crocin, which is one of the most bioactive components of saffron, has antioxidant, anti-inflammatory, and antitumor effects. The current study was aimed at investigating the possible protective effects of crocin against doxorubicin-induced nephrotoxicity to elucidate the underlying mechanism of this effect. The study included four groups, six rats in each group: normal control, crocin control, doxorubicin, and crocin/doxorubicin. Doxorubicin and crocin/doxorubicin groups received intraperitoneal injections of doxorubicin (3.5 mg/kg twice weekly for 3 weeks). Rats in the crocin control group and the crocin/doxorubicin group were treated with intraperitoneal injections of crocin (100 mg/kg body weight per day) for 3 weeks. Biomarkers of kidney function and oxidative stress as well as the abundance of mRNA for nuclear factor-*κβ* and inducible nitric oxide synthase were evaluated. In addition, the abundance of cyclooxygenase 2 and tumor necrosis factor *α* immunoreactivity was evaluated. Crocin treatment had renoprotective effects manifested by significant improvement in kidney function as well as a reduction in the abundance of biomarkers of oxidative stress markers and inflammatory mediators. In conclusion, crocin has a protective effect against doxorubicin-induced nephrotoxicity in rats by serving as an antioxidant and attenuating the expression of NF-*κ*B, iNOS, COX2, and TNF*α*.

## 1. Introduction

Doxorubicin is a member of the anthracycline family of cytotoxic antibiotics and one of the most potent and commonly used chemotherapeutic agents for the treatment of several types of cancer [1]. The antitumor activity of doxorubicin is attributed to its ability to intercalate into the DNA helix and/or bind covalently to proteins involved in DNA replication and transcription resulting in inhibition of DNA, RNA, and protein synthesis, leading ultimately to cell death [2]. Doxorubicin's cytotoxic effect on cells also involves inhibition of topoisomerase II activity to further impair transcription [3]. Oxidative damage to cell membranes, DNA, and proteins is another mechanism of action of doxorubicin [4]. Cardiotoxicity, neurotoxicity, hepatotoxicity, and nephrotoxicity are serious side effects of doxorubicin treatment. Doxorubicin causes almost irreversible kidney damage as the ability of the kidney to regenerate and heal is limited. This damage manifests as nephropathy, proteinuria, and glomerulosclerosis, and it has serious harmful effects on the entire body [3].

The detailed mechanisms of doxorubicin-induced renal damage remain unknown [5, 6]; however, several studies implicate oxidative stress [5, 7–9]. In addition, El-Moselhy and El-Sheikh suggest that oxidative stress induced by doxorubicin also stimulates the release of tumor necrosis factor *α* (TNF*α*), which would further activate multiple signaling pathways, including the nuclear factor *κ*B (NF-*κ*B) inflammatory pathway [10]. Consistent with this, several researchers demonstrated that doxorubicin increased NF-*κ*B, TNF*α*, inducible nitric oxide synthase (iNOS), and cyclooxygenase 2 (COX2) in renal tissue [11, 12]. Hence, we are in need of an adjuvant therapy for doxorubicin chemotherapy that would have both the antioxidant and anti-inflammatory effects required to protect against the renal damage associated with doxorubicin treatment.

Crocin is one of the most bioactive components of saffron (Crocus sativus L.), a monocotyledon species of the Iridaceae family. Saffron is cultivated in many areas of the world including Egypt [13]. Crocin has different pharmacological properties, including antioxidant, anti-inflammatory, and antitumor effects [14, 15]. Crocin was previously reported to have a cardioprotective effect in doxorubicin-treated rats [16, 17]. In addition, crocin attenuates the negative hematological effects of doxorubicin treatment in rats [18].

Crocin treatment also reduced nephropathy in other pathological settings, including gentamicin-induced nephrotoxicity [19], diabetes [20], carbon tetrachloride nephropathy [21], and methotrexate nephropathy [22]. The reported anti-inflammatory properties of crocin included reducing the abundance of COX2 and TNF*α* mRNA as well as iNOS expression and nitric oxide production via downregulation of NF-*κ*B activity [23]. To the best of our knowledge, the effect of crocin on doxorubicin-induced nephropathy has not been addressed before. Hence, the present study was undertaken to investigate (1) the possible protective effects of crocin on doxorubicin-induced nephrotoxicity and (2) the underlying mechanisms mediating this effect.

## 2. Materials and Methods

This work was performed at the Medical Physiology Department, Faculty of Medicine, Suez Canal University, Ismailia, Egypt.

### 2.1. Animals

Twenty-four adult male albino Sprague-Dawley rats, body weight 130-175 g, were purchased from the Egyptian Organization for Biological Products and Vaccines (Giza, Egypt). This study was performed in accordance with the Guide for the Care and Use of Laboratory Animals (1985), NIH, Bethesda, and was approved by the Ethics Committee of the Suez Canal University Faculty of Medicine. All rats were allowed to acclimatize for one week prior to the experiment and were housed in plastic cages maintained at controlled room temperature (22-24°C) with a 12-hour diurnal (day and night change) cycle and free access to tap water and a standard rat chow diet. Rats were randomly allocated to four groups: normal control, crocin control, doxorubicin, and crocin/doxorubicin groups.

### 2.2. Doxorubicin-Induced Nephrotoxicity and Crocin Treatment

Rats in the doxorubicin and crocin/doxorubicin groups received intraperitoneal injections of doxorubicin (3.5 mg/kg, doxorubicin-HCl, Pfizer, Egypt) twice weekly for 3 weeks [24]. Rats in the crocin control and crocin/doxorubicin groups received intraperitoneal injections of crocin (100 mg/kg, Sigma-Aldrich, Catalog #17304) daily [19] for 3 weeks.

### 2.3. Urine Collection and Blood Sampling

At the end of the study, 24-hour urine samples were collected to determine urine albumin and creatinine levels. Rats were then anesthetized with ether, and retrobulbar blood samples were collected and processed by centrifugation at 2000 × *g* for 15 min. Serum samples were separated, collected in clean tubes, and stored at -80°C until use. Anesthetized rats were sacrificed by decapitation, and laparotomy was done for kidney collection. Right kidneys were kept in 10% buffered formalin solution for subsequent processing for histopathological and immunohistochemical evaluation. Left kidneys were frozen and stored at -80°C prior to homogenization and subsequent assays.

### 2.4. Assay of Biochemical Markers of Kidney Function in Blood and Urine

Serum albumin, blood urea nitrogen (BUN), creatinine, and urine albumin and creatinine were determined using commercial kits on the Cobas c311 analyzer (Roche Diagnostics, Germany). The standard conventional formula (urinary creatinine concentration (U) (mg/ml) × urine volume (V) (ml/min)/serum creatinine concentration (P) (mg/ml)) was used for creatinine clearance calculation [25].

### 2.5. Assays of Oxidative Stress in Kidney Homogenate

Kidneys were homogenized in 10 volumes (*w*/*v*) of Tris buffer (10 mM Tris HCl, 1 mM EDTA, 0.32 M sucrose, pH 7.8) using a Teflon and glass homogenizer (Glas Col homogenizer system, Vernon Hills, USA). The homogenate was sonicated and then centrifuged at 20,000 × *g* for 10 min. Lipid peroxides (malondialdehyde, MDA) and superoxide dismutase (SOD) activity were assayed in renal tissue homogenate by the colorimetric method using specific kits supplied by Biodiagnostic, Egypt, according to the manufacturer's instructions.

### 2.6. Quantitative Real-Time Polymerase Chain Reaction (qPCR) for NF-*κ*B and iNOS mRNA Expression in Kidney Tissues

Total RNA was isolated from left kidney frozen tissue using the Qiagen tissue extraction kit (Qiagen, USA) according to the manufacturer's instructions. Extracted RNA was quantified by spectrophotometry (dual wavelength Beckman, spectrophotometer, USA). The RNA integrity was assessed using agarose gel electrophoresis and ethidium bromide staining. The total RNA (0.5–2 *μ*g) was used to prepare cDNA using a high fidelity reverse transcription kit (Fermentas, USA). Real-time qPCR amplification and analysis were performed using an Applied Biosystems instrument with StepOne™ software (version 3.1). Reaction mixtures contained SYBR Green Master Mix (Applied Biosystems), gene-specific forward and reverse primers (10 mM), and cDNA and nuclease-free water. The sequences of PCR primer pairs used for each gene are shown in Table 1. Cycling conditions were 10 min at 95°C followed by 40 cycles of 15 seconds at 95°C and 1 min at 60°C [26, 27].

### 2.7. Histological and Morphometric Analysis of the Kidney

Right kidneys were dissected and immediately transferred to 10% buffered formalin solution for fixation. Kidneys were dehydrated using a series of solutions of increasing ethanol content and then embedded in paraffin. Sections of 4 *μ*m thickness were cut, stained with hematoxylin and eosin (H&E), and examined with an Olympus light microscope. About 20 sections for the H&E stained kidney sections were selected for the measurement of glomerular, proximal, and distal tubular areas [28] using ImageJ. The glomerular area was assessed at ×100 magnification while proximal and distal tubular areas were assessed at ×400 magnification.

### 2.8. Immunohistochemical Evaluation of the Kidney

Kidney sections were cut, rehydrated, deparaffinized, and mounted for immunostaining. TNF*α* immunostaining was using a polyclonal rabbit anti-TNF*α* antibody diluted 1 : 400 (GeneTex, Cat# GTX110520). COX2 immunostaining used a polyclonal rabbit anti-COX2 antibody diluted 1 : 100 (GeneTex, Cat# GTX1100656). Twenty sections were evaluated for each group. The DAB-stained cytoplasmic option in the IHC profiler ImageJ plugin was used, as described by Varghese et al. [29]. Images were acquired using an Olympus microscope and camera interfaced with an IBM desktop computer. Images were acquired at ×10 glomerular areas and ×40 proximal and distal tubular areas.

### 2.9. Statistical Analysis

Parametric data were expressed as mean ± standard error of mean (SEM), and nonparametric data were presented as median and range (minimum-maximum). For comparisons of quantitative variables among the study groups, one-way ANOVA followed by the Bonferroni post hoc test was used if data were parametric, while the Kruskal-Wallis (KW) test was used if data were nonparametric. Statistical analysis was done by the Statistical Package for Social Sciences (SPSS) program version 20. Data were considered statistically significant with *p* ≤ 0.05.

## 3. Results

### 3.1. Effect of Doxorubicin and Crocin on Kidney Function Biochemical Markers

**Figure 1:**
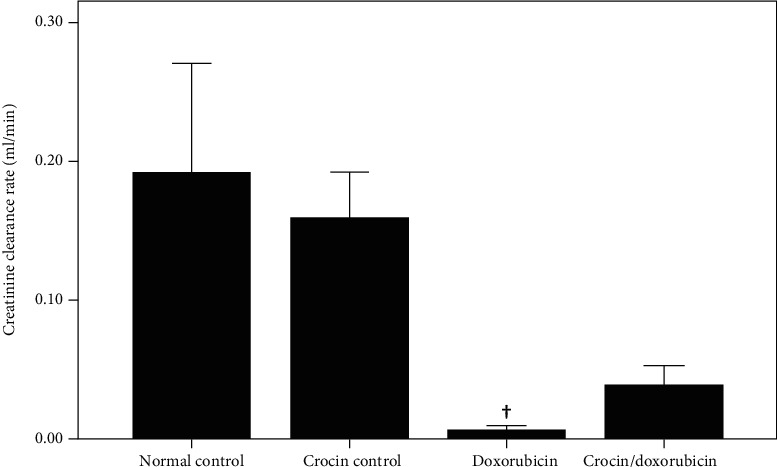
Creatinine clearance rate (mean ± SEM) in the study groups. ^†^Significant decrease in creatinine clearance rate in the doxorubicin group vs. the normal control group (*p* = 0.034).

The assessment of biochemical markers of kidney function revealed significant deterioration of kidney function in the doxorubicin group. Specifically, there was a decrease in serum albumin concentration in the doxorubicin group in comparison with both the normal control group and the crocin control group (*p* = 0.008 and *p* = 0.015, respectively), an increase in serum creatinine concentration in the doxorubicin group in comparison with the normal control group (*p* = 0.034), an increase in urine albumin concentration in the doxorubicin group in comparison with the normal control group (*p* = 0.036), a decrease in urine creatinine concentration in the doxorubicin group in comparison with both the normal control group and the crocin control group (*p* = 0.021 and *p* = 0.038, respectively), an increase in urine albumin/creatinine ratio (ACR) in the doxorubicin group in comparison with the normal control, crocin control, and crocin/doxorubicin groups (*p* = 0.004, *p* = 0.007, and *p* = 0.0007, respectively), and a decrease in the creatinine clearance rate in the doxorubicin group in comparison with the normal control group (*p* = 0.034). The biomarkers of kidney function in the crocin+doxorubicin group did not differ significantly from those of the normal control group (Table 2 and Figure 1). Hence, administration of crocin with doxorubicin attenuated the ability of doxorubicin to impair kidney function.

### 3.2. Effect of Doxorubicin and Crocin on Renal Oxidative Stress

Analysis of biomarkers of oxidative stress showed that doxorubicin caused a significant increase in MDA, a marker of lipid peroxidation compared with the normal control group (*p* = 0.017). There was also a significant decrease in the antioxidant SOD in the doxorubicin group in comparison with the normal control group and the crocin control group (*p* = 0.018 and *p* = 0.024, respectively). The administration of crocin in combination with doxorubicin decreased in MDA (*p* = 0.026) and increased SOD (*p* = 0.012) in renal tissues relative to the doxorubicin group (Table 3).

### 3.3. Effect of Doxorubicin and Crocin on NF-*κ*B and iNOS mRNA Expression in Renal Tissues

**Figure 2:**
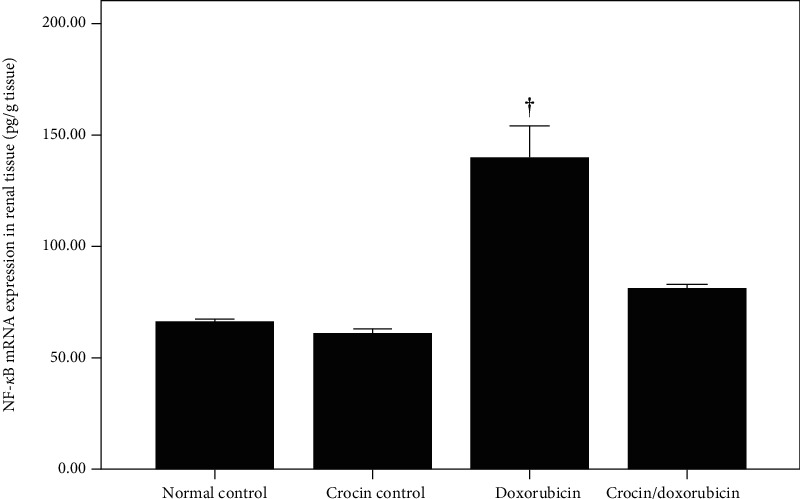
NF-*κ*B mRNA expression (mean ± SEM) in renal tissues of the study groups. ^†^Significant increase in renal NF-*κ*B mRNA expression in the doxorubicin group vs. the normal control group, the crocin group, and the crocin/doxorubicin group (*p* < 0.01 in all comparisons).

**Figure 3:**
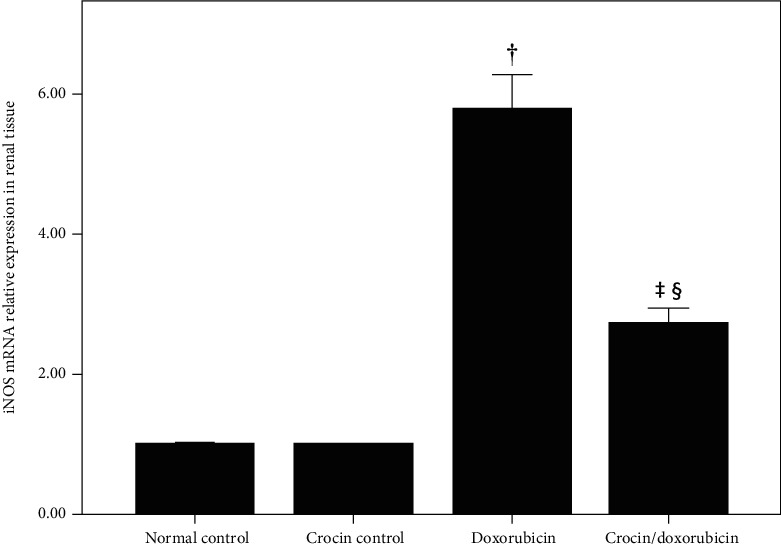
iNOS mRNA relative expression (mean ± SEM) in renal tissues of the study groups. ^†^Significant increase in renal iNOS mRNA relative expression in the doxorubicin group vs. the normal control group and the crocin control group (*p* < 0.01 in both comparisons). ^§^Significant decrease in renal iNOS mRNA relative expression in the crocin/doxorubicin group vs. the doxorubicin group (*p* < 0.01). ^‡^Significant increase in renal iNOS mRNA relative expression in the crocin/doxorubicin group vs. the normal control group and the crocin control group (*p* < 0.01 in both comparisons).

To elucidate the possible mechanism underlying the findings described above, NF-*κ*B and iNOS mRNA were quantified in renal tissue by qPCR. Doxorubicin increased the abundance of renal NF-*κ*B mRNA compared with both the normal control group and the crocin group (*p* < 0.01 for both). Crocin administration with doxorubicin decreased the abundance of NF-*κ*B mRNA relative to the doxorubicin group (*p* < 0.01) (Figure 2). The abundance of iNOS mRNA was also greater in the doxorubicin group when compared with the normal control group, crocin group, and crocin/doxorubicin group (*p* < 0.01 for all comparisons). However, although the administration of crocin plus doxorubicin decreased iNOS mRNA relative to doxorubicin alone, it remained elevated when compared with both the normal control group and the crocin group (*p* < 0.01 for both comparisons) (Figure 3).

### 3.4. Histopathological and Morphometric Analysis of the Kidney

**Figure 4:**
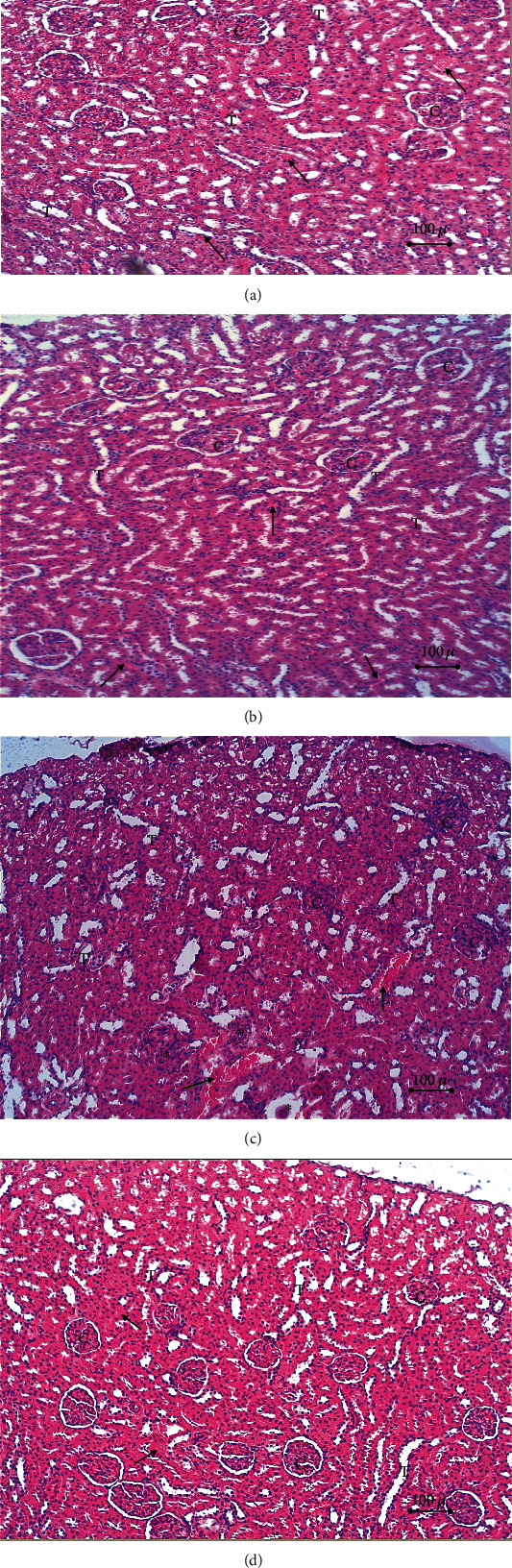
Histopathological results of the kidney in the study group. Section in the renal cortex of (a) the normal control group, (b) the crocin control group, (c) the doxorubicin group, and (d) the crocin/doxorubicin group. c: nephrogenic corpuscles; T: tubules; arrow: points to blood vessels; asterisk: points to degenerated nephrogenic corpuscles (H&E ×10).

**Figure 5:**
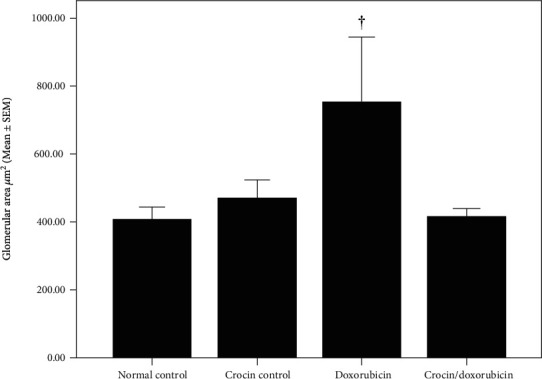
Glomerular area (*μ*m^2^) (mean ± SEM) in the study groups. ^†^Significant increase in glomerular area in the doxorubicin group vs. the normal control group and the crocin/doxorubicin group (*p* = 0.048 and 0.035, respectively).

Histopathological evaluation of sections of the renal cortex in the normal control group and the crocin control group showed normal renal corpuscle and tubule morphology. In the doxorubicin group, renal sections revealed distorted stroma with multiple vacuoles, congested and dilated blood vessels, some of the renal corpuscles were edematous with obliterated Bowman's capsules, and other corpuscles were degenerated. Some renal tubules were dilated whereas others were compressed and obliterated. In the crocin/doxorubicin group, renal sections showed reduced pathological change and preservation of normal renal cortex architecture (Figure 4). Morphometric analysis revealed a greater glomerular area in the doxorubicin group relative to both the normal control group and the crocin/doxorubicin group (*p* = 0.048 and *p* = 0.035, respectively) (Figure 5). In addition, the proximal convoluted tubule area was reduced in the doxorubicin group in comparison with the normal control, crocin control, and crocin/doxorubicin groups (*p* < 0.01, *p* = 0.046, and *p* < 0.01, respectively) (Table 4).

### 3.5. TNF*α* and Cyclooxygenase 2 Immunohistochemistry in Renal Tissues

**Figure 6:**
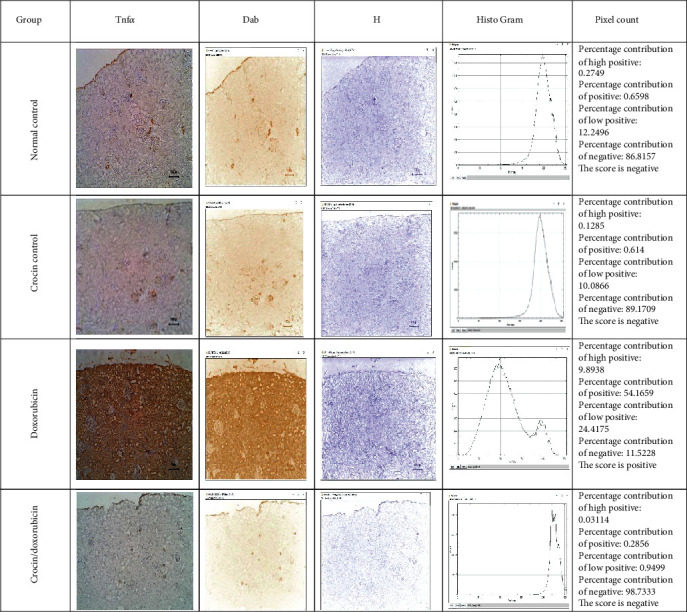
Renal TNF*α* immunostaining with ImageJ IHC profiler analysis in the study groups. Analysis of renal slides of the different groups stained with TNF*α* immunostaining using the ImageJ IHC profiler revealed that control and crocin groups gave a low negative reaction to the TNF*α* immunostaining while doxorubicin presented a positive reaction in contrast to the crocin/doxorubicin group which presented a negative reaction.

**Figure 7:**
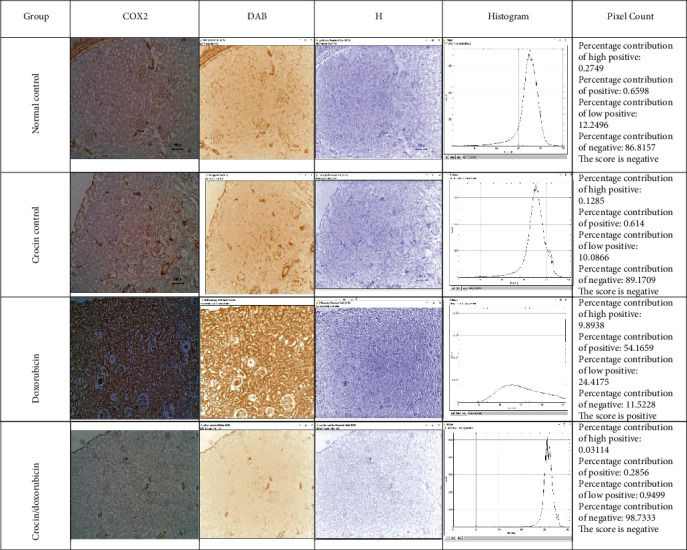
Renal COX2 immunostaining with ImageJ IHC profiler analysis in the study groups. Analysis of renal slides of the different groups stained with COX2 immunostaining using the ImageJ IHC profiler revealed that control and crocin groups gave a low positive reaction to the COX2 immunostaining while the doxorubicin group presented a positive reaction in contrast to the crocin/doxorubicin group which presented a negative reaction.

The abundance of TNF*α* and cyclooxygenase 2 immunoreactivity was assessed in renal tissue sections immunohistochemically. Immunostaining for TNF*α* was negative in both normal control and crocin control groups. In contrast, the doxorubicin group showed intense staining, which was markedly decreased in the crocin/doxorubicin group (Figure 6). Staining for COX2 revealed negligible levels in both normal control and crocin control groups, whereas the intensity of COX2 immunoreactivity was markedly increased in the doxorubicin group. Administration of crocin along with doxorubicin decreased the abundance of COX2 immunoreactive staining in comparison with that of sections from the crocin/doxorubicin group (Figure 7).

## 4. Discussion

Kidney injury is a global health problem associated with high morbidity, mortality, and healthcare costs. In addition, drug-induced nephrotoxicity is a major concern associated with the administration of chemotherapeutic agents. Anticancer therapy generally affects multiple organs, including the kidneys, leading to acute and chronic kidney diseases, renal dysfunction, and end-stage renal disease. Doxorubicin is an antitumor drug with a wide spectrum of activity in human cancers that, unfortunately, has serious side effects, including nephrotoxicity [30]. The current study investigated the protective effect of crocin treatment on doxorubicin-induced nephrotoxicity. Doxorubicin administration caused significant deterioration of kidney function and marked histopathological changes, as reported previously [24, 31], that were attenuated by coadministration of crocin.

In this study, the mechanisms underlying doxorubicin-induced nephrotoxicity were addressed by evaluating the renal markers of oxidative stress markers and the expression of NF-*κ*B, iNOS, COX2, and TNF*α*. MDA, a biomarker of lipid peroxidation, was increased, and SOD was decreased, indicating that doxorubicin treatment induced an oxidant-antioxidant imbalance in renal tissue. This finding agrees with several previous studies [6, 11]. It has been suggested that the increase of reactive oxygen species leads to activation of NF-*κ*B which, in turn, leads to the induction of key inflammatory mediators including iNOS, TNF*α*, and COX2. The increase in these proinflammatory mediators in turn leads to tissue injury and further activation of NF-*κ*B. This positive feedback mechanism is believed to amplify inflammatory signals and exacerbate tissue injury [32].

To the best of our knowledge, the findings of the current study demonstrate for the first time that crocin has a protective effect against doxorubicin-induced nephrotoxicity in rats by preserving renal structure and function. Furthermore, our findings suggest that the renoprotective effects of crocin may be attributed to prevention of the doxorubicin-induced increase in NF-*κ*B, iNOS, COX2, and TNF*α* expression resulting in a reduction in oxidative stress in the kidneys.

The observed antioxidant effect of crocin in renal tissues is consistent with the results of previous studies showing crocin inhibited the increase in lipid peroxidation induced by cisplatin in renal tissue [33]. Crocin supplementation has also been shown to ameliorate the renal oxidant/antioxidant imbalance induced by advancing age in rats [14]. Finally, by preserving the oxidant-antioxidant balance, crocin prevented methotrexate-induced renal damage [22].

All inflammatory disorders are associated with a release of reactive oxygen species with proinflammatory molecules [13]. And the anti-inflammatory effect of crocin is based on its antioxidant and free radical scavenging properties. Previous studies have reported that crocin treatment significantly reduced the abundance of mRNA for proinflammatory mediators interleukin-6 and TNF*α* in the kidneys of aged rats [14]. Crocin treatment has also been shown to slow the progression of diabetic nephropathy by modulating the oxidative burden and the inflammatory cascade [20]. Finally, crocin prevented the increase in intercellular adhesion molecule-1 and TNF*α* mRNA associated with ischemia-reperfusion induced renal injuries in rats [34].

It is known that NF-*κ*B regulates the expression of iNOS, COX2, and proinflammatory cytokines at the transcriptional level [35]. Interestingly, we found that crocin downregulated the doxorubicin-induced increase in NF-*κ*B mRNA, which in turn attenuated the increase in iNOS mRNA as well as COX2 and TNF*α* immunoreactivity in renal tissues. Consistent with these observations, crocin was shown to decrease the protein levels of the NF-*κ*B p65 subunit in the hippocampus of lipopolysaccharide-treated mice [36] and reduce the expression of NF-*κ*B and consequently inhibit the downstream inflammatory cascade manifested by decreasing the expression of COX2 and levels of TNF*α* and IL-1*β* in thioacetamide-induced liver fibrosis in mice [37].

Interestingly, despite the ability of crocin to prevent the doxorubicin-induced increase in NF-*κ*B mRNA, the abundance of iNOS mRNA was significantly higher in the crocin/doxorubicin group than observed in the normal control group. However, it was previously reported that in most in vitro studies, only a combination of multiple cytokines was able to elicit a profound increase in iNOS mRNA, whereas a single stimulus exhibited only a moderate effect in specific cell types. These observations suggest that two or more signal transduction pathways are necessary to fully upregulate iNOS expression. One important intracellular signal transduction pathway is the activation of NF-*κ*B. Alternative pathways include the Janus tyrosine kinase- (JAK-) signal transducers and activators of transcription (STAT). In addition, the mitogen-activated protein kinase (MAPK) pathway most likely contributes to iNOS gene expression [38]. Hence, we suggest that doxorubicin increased the abundance of iNOS mRNA through the activation of two or more signal transduction pathways: the NF-*κ*B represents one of these pathways and is inhibited by crocin. Hence, the crocin-mediated inhibition of the ability of doxorubicin to increase NF-*κ*B mRNA resulted in a partial decrease in doxorubicin-induced iNOS expression while the other doxorubicin-activated pathways also leading to iNOS upregulation were not targeted by crocin. A limitation of the current study is that the observed changes in the renal expression of NF-*κ*B and iNOS have not yet been confirmed at the protein level.

In conclusion, the present study shows that, as an adjuvant therapy for doxorubicin chemotherapy, crocin has a renoprotective effect in that it attenuated doxorubicin-induced nephrotoxicity in rats by serving as an antioxidant and suppressing the doxorubicin-induced increase in NF-*κ*B, iNOS, COX2, and TNF*α*. Further studies are required to evaluate other possible mechanisms mediating the renoprotective effects of crocin and to address the safety of using crocin/doxorubicin cotreatment in humans.

## Figures and Tables

**Table 1:** Primer sequence used for qPCR.

Primer	Sequence
NF-*κ*B	Forward CAGCTCTTCTCAAAGCAGCA
	Reverse TCCAGGTCATAGAGAGGCTCA
iNOS	Forward CTTTGCCACGGACGAGAC
	Reverse TCATTGTACTCTGAGGGCTGAC

**Table 2:** Kidney function biochemical markers: BUN and serum albumin and creatinine concentration, urine albumin and creatinine concentration, and urine albumin creatinine ratio (ACR) (mean ± SEM) in the study groups.

	BUN (mg/dl)	Serum albumin (mg/dl)	Serum creatinine (mg/dl)	Urine albumin (mg/dl)	Urine creatinine (mg/dl)	ACR (mg/g)
Normal control group	14.5 ± 0.5	3.37 ± 0.13	0.22 ± 0.09	0.1 ± 0.02	28.18 ± 8.1	2.98 ± 0.49
Crocin control group	13.45 ± 1.45	3.23 ± 0.13	0.38 ± 0.08	0.40 ± 0.37	28.85 ± 5.91	18.23 ± 5.78
Doxorubicin group	18 ± 1.47	1.93 ± 0.22^†^	0.57 ± 0.03^‡^	0.64 ± 0.14^‡^	2.83 ± 1.28^§^	926.85 ± 319.32^¶^
Crocin/doxorubicin group	19.27 ± 3.24	2.37 ± 0.35	0.46 ± 0.03	0.51 ± 0.23	13.46 ± 4.59	85.53 ± 14.24

^†^Significant decrease in serum albumin concentration in the doxorubicin group vs. the normal control group and the crocin control group (*p* = 0.008 and 0.015, respectively). ^‡^Significant increase in serum creatinine concentration in the doxorubicin group vs. the normal control group (*p* = 0.034) and significant increase in urine albumin concentration in the doxorubicin group vs. the normal control group (*p* = 0.036). ^§^Significant decrease in urine creatinine concentration in the doxorubicin group vs. the normal control group and the crocin control group (*p* = 0.021 and 0.038, respectively). ^¶^Significant increase in urine ACR in the doxorubicin group vs. the normal control, crocin control, and crocin/doxorubicin groups (*p* < 0.01 in all comparisons).

**Table 3:** Oxidative stress marker concentration (mean ± SEM) in the renal tissues in the study groups.

	MDA (nmol/g tissue)	SOD (*μ*/g tissue)
Normal control group	17.47 ± 3.93	3.48 ± 0.08
Crocin control group	33.36 ± 2.26	3.33 ± 0.08
Doxorubicin group	76 ± 21.13^†^	1.03 ± 0.68§
Crocin/doxorubicin group	22.59 ± 2.85^‡^	3.5 ± 0.013

^†^Significant increase in MDA concentration in renal tissues in the doxorubicin group vs. the normal control group (*p* = 0.017). ^‡^Significant decrease in MDA concentration in renal tissues in the crocin/doxorubicin group vs. the doxorubicin group (*p* = 0.026). ^§^Significant decrease in SOD concentration in renal tissues in the doxorubicin group vs. the normal control group, the crocin control group, and the crocin/doxorubicin group (*p* = 0.018, 0.024, and 0.012, respectively).

**Table 4:** Area of renal proximal convoluted tubules and distal convoluted tubules *μ*m^2^ (median, minimum, and maximum) in the study groups.

	Area of renal proximal convoluted tubules	Area of renal distal convoluted tubules
Normal control group	6173.13 (3811.22-16503.69)	4433.99 (1102.16-11303.81)
Crocin control group	6857.18 (3870.87-12009.27)	5013.93 (3234.47-10969.46)
Doxorubicin group	3543.64 (1347.17-8035.63)^∗^	3966.79 (1331.53-14499.75)
Crocin/doxorubicin group	7015.2 (2312.6-14123.54)	5575.57 (664-13746.85)

^∗^Significant decrease in the area of proximal convoluted tubules in the doxorubicin group vs. the normal control, crocin control, and crocin/doxorubicin groups (*p* < 0.01, *p* = 0.046, and *p* < 0.01, respectively).

## Data Availability

The data used to support the findings of this study are available from the corresponding author upon request.

## Additional Points

*Key Points*. (1) Crocin has a renoprotective effect on doxorubicin-induced nephrotoxicity. (2) Crocin inhibits renal expression of NF-*κ*B, iNOS, COX2, and TNF*α* in nephrotoxicity.
